# Supplementary material for: The interactome of intact mitochondria by cross-linking mass spectrometry provides evidence for coexisting respiratory supercomplexes
Source: Mol Cell Proteomics. 2017 Dec 8;17(2):216–32. doi: 10.1074/mcp.RA117.000470 (PMC5795388; doi:10.1074/mcp.RA117.000470)
Supplement: Supplemental Data [file supp_RA117.000470_133922_0_supp_23978_fzffwf.zip › spectra_annotation/mito_spectra_annotation/472-1-3-1-1-1.pdf]

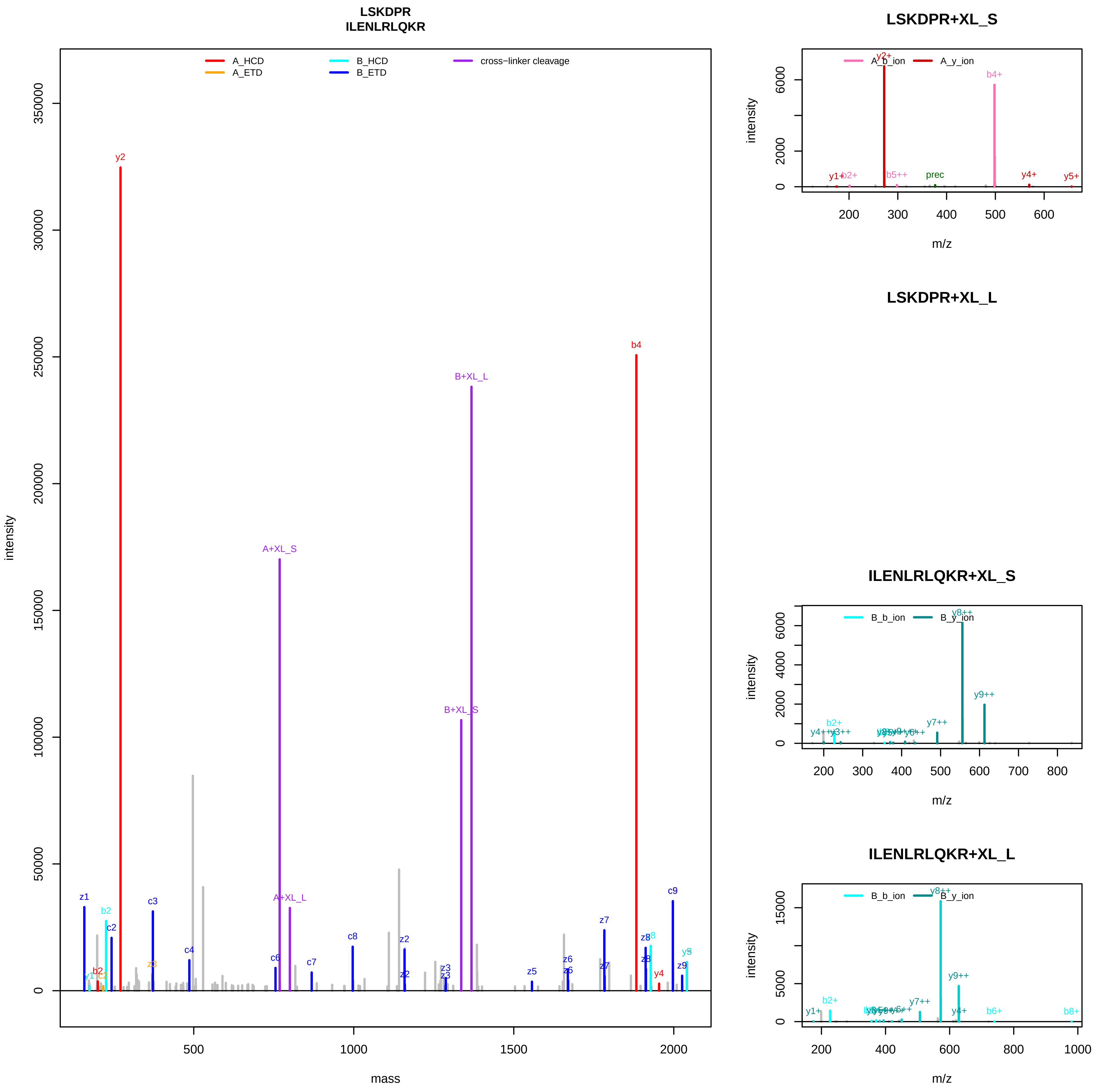
LSKDPR
ILENLRLQKR
A_HCD
A_ETD
B_HCD
B_ETD
cross−linker cleavage
intensity
mass
y2
b4
B+XL_L
A+XL_S
B+XL_S
A+XL_L
z1
b2
c2
c3
c4
c6
c7
c8
z2
z3
z5
z6
z7
z8
y4
c9
z9
LSKDPR+XL_S
A_b_ion
A_y_ion
y2+
b4+
y1+
b2+
b5++
prec
y4+
y5+
m/z
LSKDPR+XL_L
ILENLRLQKR+XL_S
B_b_ion
B_y_ion
y8++
y9++
y7++
y4++
y3++
ILENLRLQKR+XL_L
y1+
b6+
b8+
